# Structure and evolution of the embryonic cartilaginous skull of crocodilians

**DOI:** 10.1098/rsos.251281

**Published:** 2025-11-05

**Authors:** María Victoria Fernandez Blanco, Ingmar Werneburg

**Affiliations:** ^1^ División Paleontología Vertebrados, Unidades de Investigación Anexo II Museo, Museo de La Plata, Facultad de Ciencias Naturales y Museo, Universidad Nacional de La Plata, Calles 122 y 60, B1900FWA, La Plata, Buenos Aires, Argentina; ^2^ Consejo Nacional de Investigaciones Científicas y Técnicas (CONICET), Godoy Cruz 2290, C1425FQB, Ciudad Autónoma de Buenos Aires, Argentina; ^3^ Fachbereich Geowissenschaften der Eberhard Karls Universität Tübingen, Tübingen, Germany; ^4^ Senckenberg Centre for Human Evolution and Palaeoenvironment (SHEP) an der Universität Tübingen, Tübingen, Germany

**Keywords:** three-dimensional reconstruction, *Caiman crocodilus*, chondrocranium, comparative anatomy, cranial morphological diversity, embryonic development, extant crocodilian species, tempus optimum

## Abstract

The evolution of skull diversity in Crocodylia is rather well documented, but the developmental foundation of their cranial architecture remains poorly understood. Here, we present the first three-dimensional reconstruction of the embryonic cartilaginous skull of *Caiman crocodilus* based on histological sections. We provide a comprehensive anatomical description and morphometric analysis of the chondrocranium of this species, integrating linear measurements and comparative anatomical data to assess interspecific variation and evaluate whether closely related taxa exhibit greater similarity in chondrocranial morphology. We identified both qualitative and quantitative differences between major crocodilian clades, which may reflect diverse ecological demands. Within Crocodylidae, orbitotemporal proportions strongly influence chondrocranial morphology, likely reflecting adaptations related to bite force and visual acuity, linked to prey type and diverse aquatic habits. Within Alligatoridae, by contrast, the emphasis on nasal capsule proportions suggests a reliance on olfactory and respiratory functions, consistent with their occupation of densely vegetated environments and more restricted geographic ranges. Additionally, we identified a set of embryonic diagnostic features located in the splanchnocranium of *Caiman* and in the neurocranium of Alligatoridae. These findings shed new light on the developmental basis of cranial diversity in Crocodylia and emphasize the evolutionary significance of chondrocranial traits in shaping macroevolutionary patterns.

## 1. Introduction

During early vertebrate development, the initial cartilaginous structure of the skull―known as the chondrocranium―forms the foundation of the cranial skeleton. In some groups, such as chondrichthyans and many squamates, this structure remains entirely or partially cartilaginous throughout life. In others, including reptiles, birds and mammals, it progressively ossifies, contributing to the formation of the bony skull [1–4]. The extent and pattern of this ossification vary among taxa, with different regions undergoing ossification at distinct ontogenetic stages. Once fully formed―referred to as tempus optimum (*sensu* [5])―the chondrocranium provides structural support to the skull and serves as a platform for the formation of both membrane bones (formed by direct ossification in the surrounding mesenchyme) and endochondral bones (formed by the replacement of cartilage) [6,7].

As an early, foundational structure that precedes the ossified skull, the chondrocranium provides the initial scaffolding for the neurocranium, splachnocranium and related skeletal elements, and it plays a key role in shaping the morphology and spatial organization of major cranial components (e.g. [2,4,8–12]). Therefore, its anatomical features can offer a valuable window into the developmental processes and morphological transformations that underpin skull evolution in vertebrates, reflecting broader evolutionary–developmental (evo–devo) dynamics that contribute to cranial diversity. As such, it encodes signals of species-specific cranial architecture, offering valuable insights into both conserved developmental patterns and lineage-specific evolutionary innovations. Moreover, variations in chondrocranial morphology may mirror shifts driven by functional demands, biomechanical performance or ecological adaptations [2–4,13,14]. For instance, the modification (or even reduction) of certain cartilaginous elements, such as the nasal septum, may significantly impact the adult cranial configuration and be directly tied to functional demands such as feeding [15–17, and references therein]. Thus, by documenting chondrocranial anatomy across species, we can trace back both developmental constraints and evolutionary changes that have driven skull diversification. In addition, these data have the potential to reveal how subtle developmental shifts may trigger major morphological transformations when analysed in a phylogenetic framework, ultimately shaping large-scale evolutionary trends. However, despite its huge evolutionary and developmental significance, the chondrocranium remains poorly studied in many lineages—particularly among reptiles—leaving major gaps in our understanding of skull evolution.

Crocodilians, as the living sister group to birds and non-avian dinosaurs, are uniquely positioned to shed light on the evolutionary and developmental origins of cranial structures in Archosauria. Their relatively conservative cranial morphology, along with a less modified pattern of cranial development compared to birds, makes them particularly valuable for reconstructing early stages of skull evolution. While adult skull morphology in crocodilians has been broadly studied (e.g. [18–20]), much less is known about the embryonic origins of cranial diversity and the development of species-specific features. This gap is especially significant given recent evidence showing that many of these traits emerge during late embryogenesis, highlighting the importance of examining earlier developmental stages to fully understand how ontogeny shapes adult morphology [21]. Despite this, only a few of the 26 living crocodilian species currently recognized by the IUCN SSC Crocodile Specialist Group [22] have been investigated in terms of chondrocranial development and anatomy. As a result, the current available data remain limited (see Klembara [23] and Fernandez Blanco [24] for a comprehensive review), leaving much of the group’s morphological diversity and phylogenetic relevance unexplored. Living crocodilians can be grouped into three major clades: Alligatoridae (Alligatorinae + Caimaninae), Crocodylidae (Osteolaeminae + Crocodylinae) and Gavialidae [25]. Understanding the chondrocranial variation among these clades is crucial for identifying both conserved and divergent developmental features across crocodilians. In this study, we provide the first three-dimensional reconstruction of the chondrocranium of *Caiman crocodilus* based on histological sections. We assess its morphology in a comparative framework, combining anatomical descriptions and principal component analysis (PCA) to explore interspecific variation in chondrocranial anatomy and proportions across seven crocodilian species. We test the hypothesis that closely related taxa exhibit more similar chondrocranial morphotypes, interpreting the observed variation in light of phylogenetic, functional and ecological factors. Our findings enhance our understanding of the developmental origins of skull diversity and reinforce the central role of the chondrocranium in vertebrate evo–devo research.

## 2. Material and methods

### 2.1. Three-dimensional reconstruction and anatomical descriptions

We analysed 1187 histological sections from the head of a *Caiman crocodilus* embryo, housed in the herpetological collection of Phyletisches Museum Jena, Germany (collection number: Rept 1181; i.e. from the previous collection of Prof. Dr Dietrich Starck, Frankfurt am Main, Germany). The specimen was stained with Bouin’s solution and sectioned transversely into 12 µm thick slices. The head measures 14 mm in total length, and while the precise embryonic stage is uncertain, we consider the embryo to be within the tempus optimum [5]. The tempus optimum represents the ‘ideal’ stage in the pre-hatching development for anatomical descriptions, as the cartilaginous skull is fully developed, and ossification has not yet begun (or there are barely traces of bones that do not affect the shape of the cartilage) [5]. Each section was examined and photographed using an Olympus BH2 microscope equipped with a Canon EOS 650D digital camera at ×1 magnification. Since the dimensions of most sections exceeded the microscope’s field of view, one to five images were captured per section. These images were then merged into a single composite using the *photomerge* tool in Photoshop CS6 (v. 13.0), creating a complete image for each slice. The slices were subsequently converted to greyscale and resized (*batch* tool) using IrfanView 64 (v. 4.62). Finally, the processed images were imported into Avizo (v. 8.1.1) and aligned to generate the three-dimensional model of the chondrocranium. The manual selection tool was used to segment all cartilages.

Following the definition of de Beer [2], we described the two functional components of the cartilaginous skull, the neurocranium and splanchnocranium. We described the three compartments of the neurocranium―ethmoidal, orbitotemporal and otic-occipital region―following the morphological divisions made by previous authors studying crocodilian species [23,24,26]. For the splachnocranium (= viscerocranium), the mandibular, hyoid and branchial arches were described. Most anatomical terms follow de Beer [2], with a few (e.g. palatoquadrate, cupola anterior, epiphanial foramen) taken from Klembara [23] and Fernandez Blanco [24]. Since the chondrocranium of *Ca. crocodilus* is fully developed (i.e. tempus optimum), it was not possible to trace back the origin of the individual components that merged to form its current configuration. As the boundaries of the original elements are transitional, they are described here based on their topographic location. Interpretations regarding their origins or primary homologies were beyond the scope of this article.

### 2.2. Quantitative analyses

**Figure 1.**
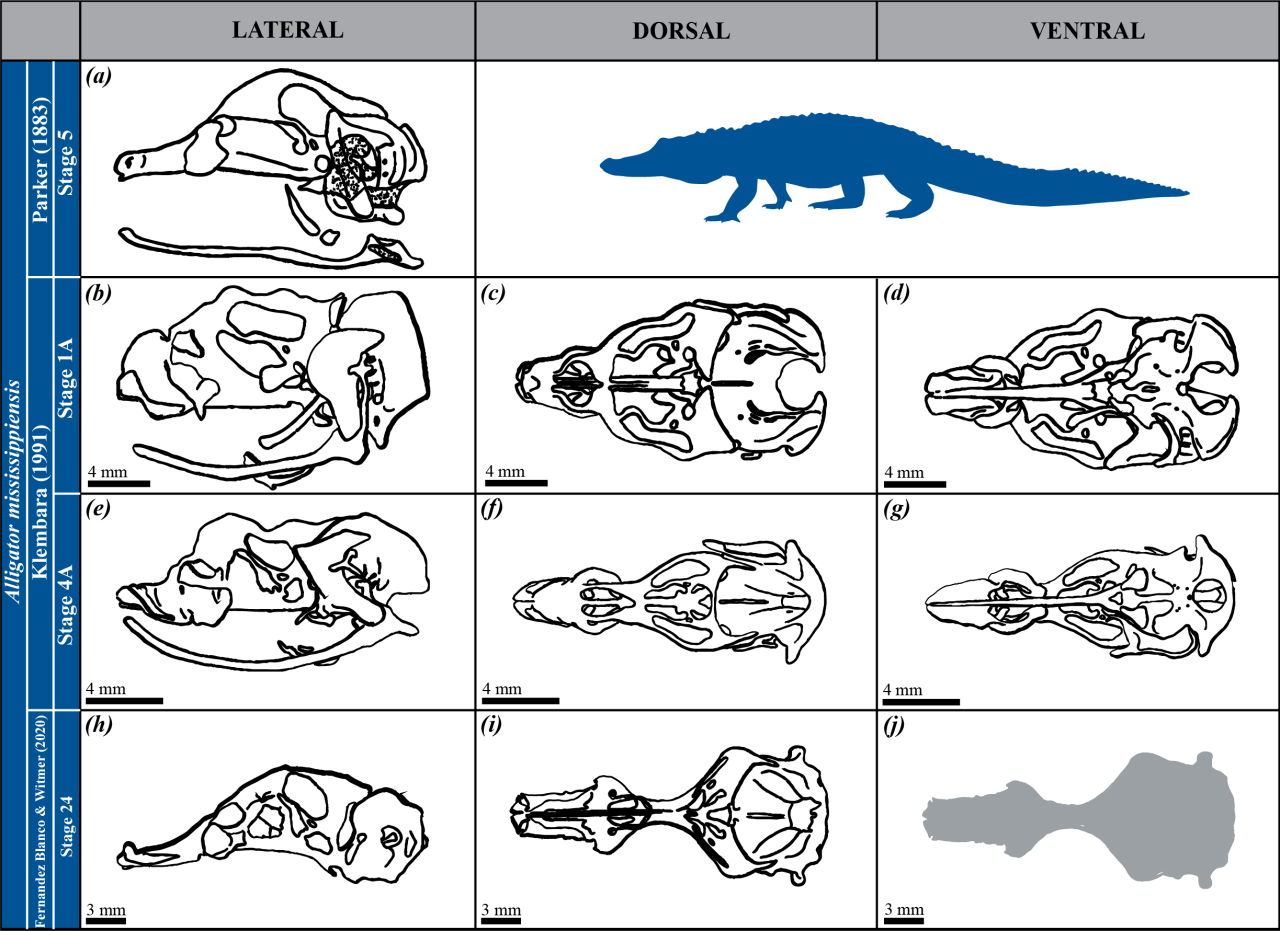
Drawings of the chondrocrania of alligatorine species (i.e. *Alligator mississippiensis*) as studied in the literature. Developmental stages, authors and publication years are indicated. Lateral (*a*,*b*,*e*,*h*), dorsal (*c*,*f*,*i*) and ventral (*d*,*g*,*j*) views are provided when available. No scale information was available in the original reference for image (*a*).

**Figure 2.**
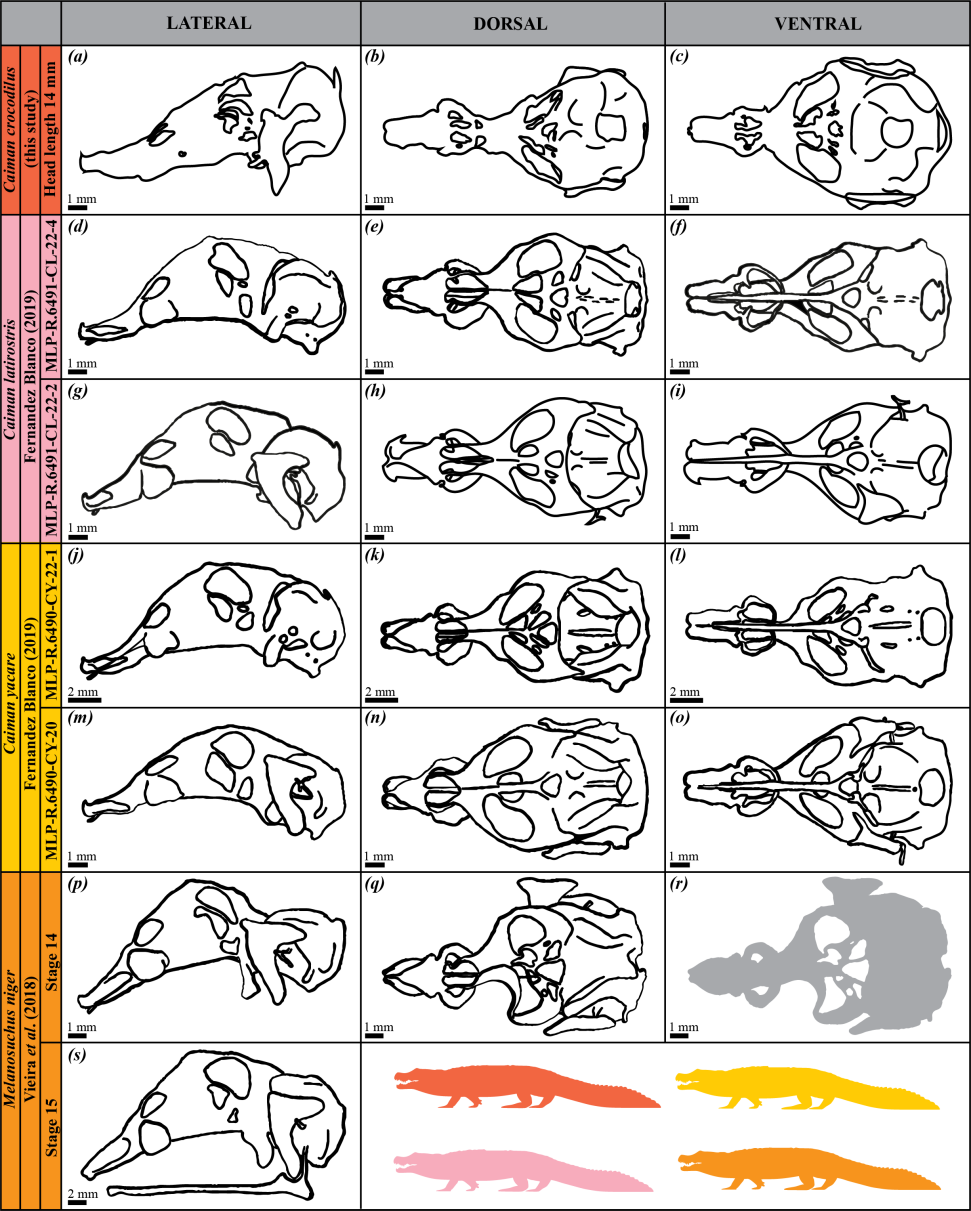
Drawings of the chondrocrania of caimanine species as studied in the literature. Specimens of *Caiman latirostris*, *Caiman yacare*, *Caiman crocodilus* and *Melanosuchus niger* are shown, with developmental stages, authors and publication years indicated. Lateral (*a*,*d*,*g*,*j*,*m*,*p*,*s*), dorsal (*b*,*e*,*h*,*k*,*n*,*q*) and ventral (*c*,*f*,*i*,*l*,*o*,*r*) views are provided when available.

**Figure 3.**
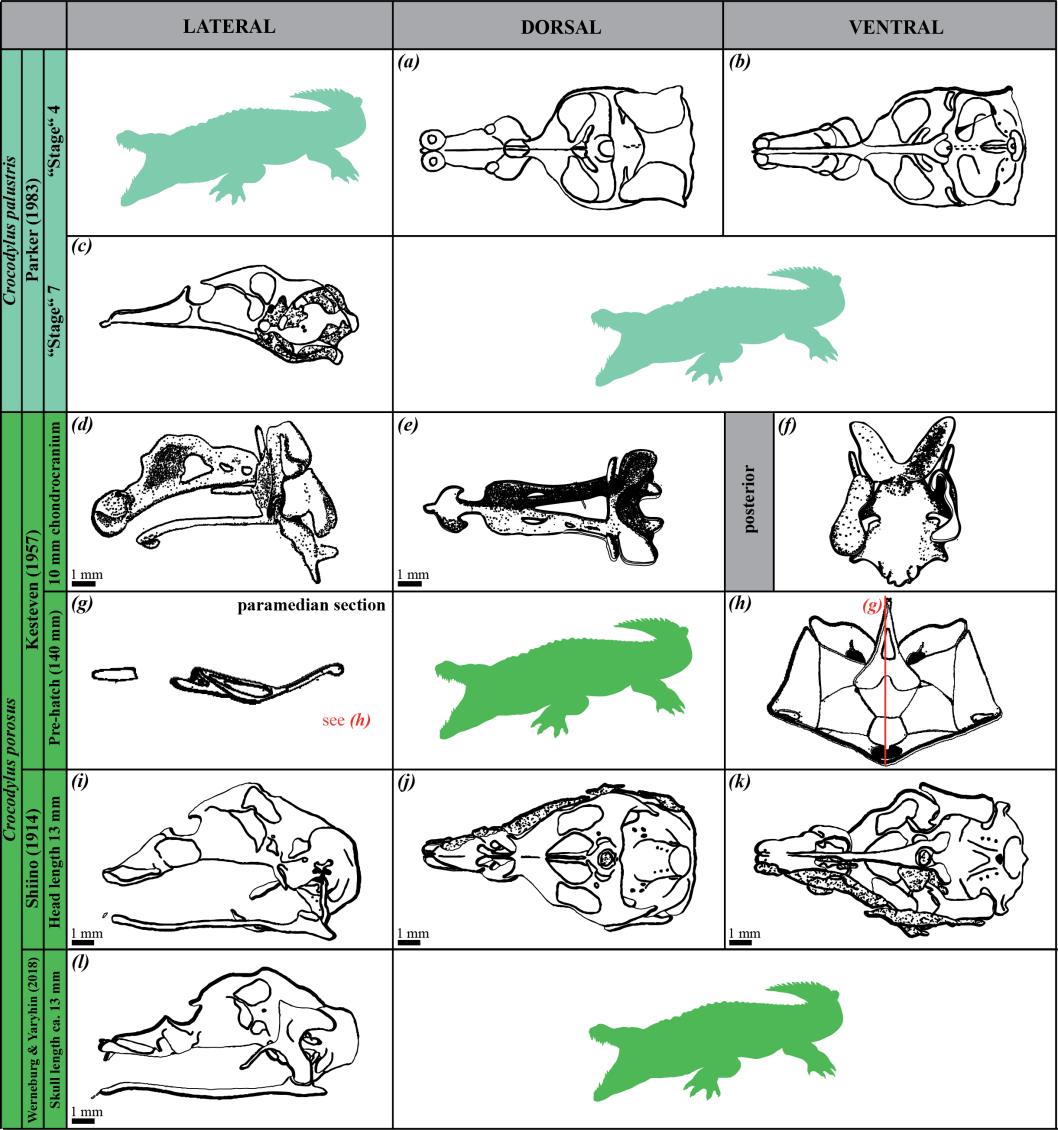
Drawings of the chondrocrania of crocodyline species (i.e. *Crocodylus porosus* and *Crocodylus palustris*) as studied in the literature. Developmental stages, authors and publication years are indicated. Lateral (*c*,*d*,*g*,*i*,*l*), dorsal (*a*,*e*,*j*) and ventral (*b*,*f*,*h*,*k*) views are provided when available. For some images, no scales were available in the original references.

**Figure 4.**
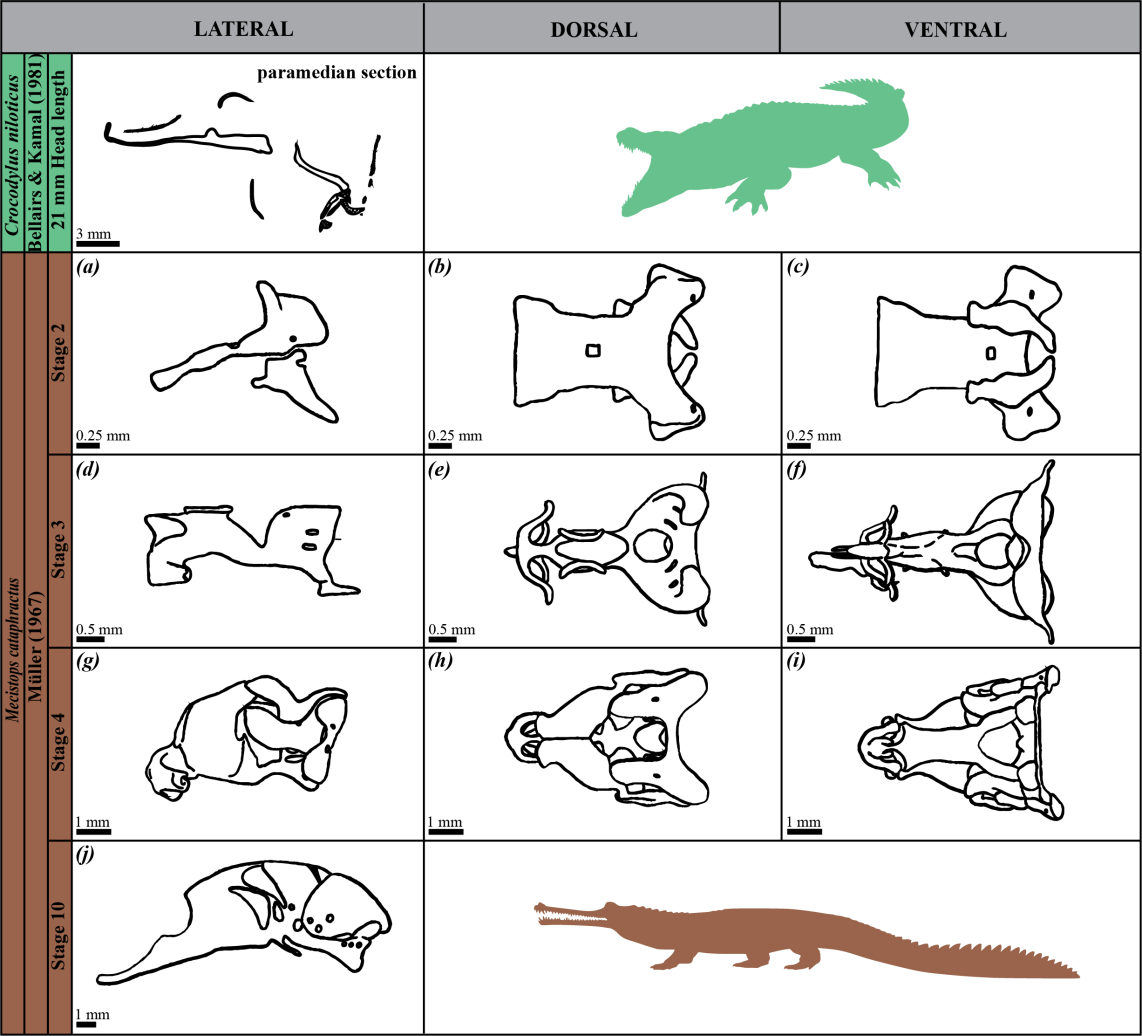
Drawings of the chondrocrania of crocodyline species (i.e. *Crocodylus niloticus* and *Mecistops cataphractus*) as studied in the literature. Developmental stages, authors and publication years are indicated. Lateral (*a*,*d*,*g*,*j*), dorsal (*b*,*e*,*h*) and ventral (*c*,*f*,*i*) views are provided when available.

We conducted a comparative study of the chondrocrania by taking 25 linear measurements from images of seven extant crocodilian species (one alligatorine, four caimanines and two crocodylines). The images were sourced from both our three-dimensional model (*Ca. crocodilus*) and existing literature, including two specimens each of *Alligator mississippiensis*, *Caiman latirostris* and *Caiman yacare*, and one specimen each of *Melanosuchus niger*, *Crocodylus palustris* and *Crocodylus porosus* (figures 1–3; table 1). All specimens were selected based on the tempus optimum definition by Werneburg & Yaryhin [5]. Although embryonic stages varied across species and were sometimes unknown, we determined that the selected specimens were within or close to the tempus optimum—the preferred period for describing the fully formed chondrocranium, as bones are not completely differentiated and, therefore, they do not alter the shape of the chondrocranium yet. Some specimens were not included because they did not fall within the tempus optimum stage (figure 4; table 1).

**Table 1.** References to crocodilian chondrocrania in the literature considered in this study, detailing the studied species, authors and years of publication, and figures and chondrocranial views used for measurements. *Indicates specimen within tempus optimum [5].

species	references		figures	views	measurements
Alligator mississippiensis*	[27]		plate 65-figure 8	lateral	no
*Alligator mississippiensis* (stage 1A)	[23]		plate 8	dorsal	no
			plate 9	ventral	
			plate 6	lateral	
Alligator mississippiensis*	[23]		plate 3	dorsal	yes
			plate 4	ventral	
			plate 1	lateral	
Alligator mississippiensis*	[28]		figure 3c	dorsal	yes
			figure 3d	lateral	
Caiman latirostris*	MLP-R.6491-CL-22-4	[24]	figure 1c	lateral	yes
		own photos		dorsal and ventral	
Caiman latirostris*	MLP-R.6491-CL-22-2 (own photos)			dorsal, ventral and lateral	yes
Caiman yacare*	MLP-R.6490-CY-22-1	[24]	figure 1a	dorsal	yes
			figure 1b	ventral	
		own photos		lateral	
Caiman yacare*	MLP-R.6490-CY-20 (own photos)			dorsal, ventral and lateral	yes
Melanosuchus niger*	[26]		figure 1g	dorsal	yes
			figure 1h	lateral	
Melanosuchus niger*	[26]		figure 3a	lateral	no
*Crocodylus palustris* (stage 7)	[27]		plate 69-figure 8	lateral	no
Crocodylus palustris*	[27]		plate 65-figure 1	dorsal	yes
			plate 65-figure 2	ventral	
Crocodylus porosus*	[29]		plate 15-figure 1	dorsal	yes
			plate 16-figure 2	ventral	
			plate 18-figure 4	lateral	
Crocodylus porosus*	[30]		figure 1b	lateral	no
*Crocodylus porosus*	[31]		figure 1	lateral	no
Crocodylus niloticus*	[3]		figure 85a	lateral	no
*Mecistops cataphractus* (stage 2)	[32]		figure 18b	dorsal	no
			figure 18c	ventral	
			figure 18a	lateral	
*Mecistops cataphractus* (stage 3)	[32]		figure 19b	dorsal	no
			figure 19c	ventral	
			figure 19a	lateral	
*Mecistops cataphractus* (stage 4)	[32]		figure 21b	dorsal	no
			figure 21c	ventral	
			figure 21a	lateral	
Mecistops cataphractus*	[32]		figure 26c	lateral	no

**Figure 5.**
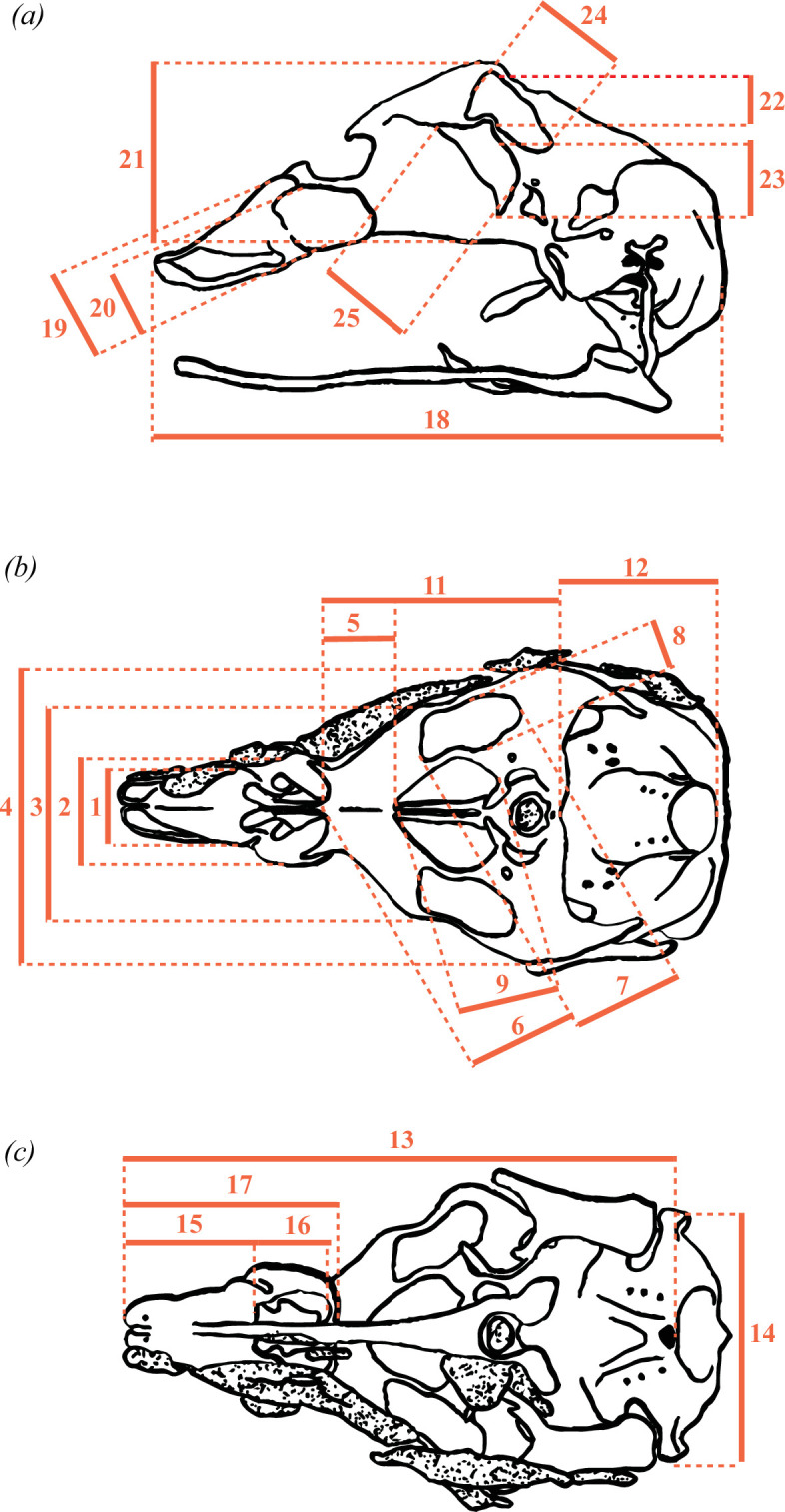
Linear measurements selected to describe morphological variation in crocodilian chondrocrania. Left lateral (*a*), dorsal (*b*) and ventral (*c*) views of the chondrocranium of *Crocodylus porosus* (adapted from Shiino [29]).

Measurements were taken from dorsal, ventral and lateral views of the chondrocrania (figure 5; table 2; electronic supplementary material, table S1) using ImageJ (v. 1.53t). Measurements were recorded in millimetres for images with scales and in pixels for *Cr. palustris*, which lacked reference scaling. These measurements were used to calculate 27 chondrocranial proportions (table 3), representing overall skull shape variation. For *A. mississippiensis* [28] and *Me. niger* [26], only dorsal and lateral views were available (figures 1 and 2; table 1); in these cases, ventral measurements were extrapolated from the dorsal view, as we considered the differences between these views to be negligible.

**Table 2.** Description of linear measurements in dorsal, ventral and lateral views of the chondrocrania.

Chondrocranial view	Descriptions of measurements
dorsal	**1.** Maximum width of the nasal capsule at the level of parietotectal cartilage
	**2.** Maximum width of the nasal capsule at the level of paranasal cartilage
	**3.** Maximum width of the chondrocranium at the level of the planum supraseptale
	**4.** Maximum width of the chondrocranium at the level of taeniae marginalis
	**5.** Length of the planum supraseptale from its anterior and medial point to the most anterior point of the optic fenestra
	**6.** Length of the planum supraseptale from its anterior and medial point to the most anterior point of the epioptic fenestra
	**7.** Maximum length of the epioptic fenestra
	**8.** Maximum width of the epioptic fenestra
	**9.** Maximum length of the optic fenestra
	**10.** Maximum width of the optic fenestra
	**11.** Maximum length of the orbitotemporal region, in the midline, from the anterior and medial point of the planum supraseptale to the crista sellaris
	**12.** Maximum length of the otic-occipital region, in the midline, from the crista sellaris to the most posterior margin of the tectum
ventral	**13.** Maximum length of the chondrocranium from the most anterior point of the nasal capsule to the occipital condyle, parallel to the midline
	**14.** Maximum width of the otic-occipital region at the level of crista parotica
	**15.** Maximum length of the nasal capsule at the level of parietotectal cartilage from the tip of the nasal capsule to the posterior border of the lamina transversalis anterior, parallel to the midline
	**16.** Maximum length of the nasal capsule at the level of paranasal cartilage from the posterior border of the lamina transversalis anterior to the lamina orbitonasalis, parallel to the midline
	**17.** Total length of the nasal capsule from its anterior tip to the lamina orbitonasalis, parallel to the midline
lateral	**18.** Total length of the chondrocranium from the anterior tip of the nasal capsule to the most posterior margin of the auditory capsule, perpendicular to the sagittal plane
	**19.** Maximum height of the parietotectal cartilage, perpendicular to its ventral edge
	**20.** Maximum height of paranasal cartilage, perpendicular to its ventral edge
	**21.** Maximum height of the chondrocranium from the most dorsal margin of the planum supraseptale to the ventral margin of the interorbital septum, perpendicular to the ventral margin of the interorbital septum
	**22.** Maximum height of epioptic fenestra
	**23.** Maximum height of optic fenestra
	**24.** Maximum length of the epioptic fenestra
	**25.** Maximum length of the optic fenestra

**Table 3.** Description of chondrocranial proportions.

**15/17**	maximum length of the nasal capsule at the level of parietotectal cartilage from the tip of the nasal capsule to the aditus conchae (anterior part), parallel to the midline/total length of the nasal capsule from its anterior tip to the lamina orbitonasalis, parallel to the midline
**16/17**	maximum length of the nasal capsule at the level of paranasal cartilage from the aditus conchae (anterior part) to the lamina orbitonasalis, parallel to the midline/total length of the nasal capsule from its anterior tip to the lamina orbitonasalis, parallel to the midline
**17/13**	total length of the nasal capsule from its anterior tip to the lamina orbitonasalis, parallel to the midline/maximum length of the chondrocranium from the most anterior point of the nasal capsule to the occipital condyle, parallel to the midline
**21/18**	maximum height of the chondrocranium from the most dorsal margin of the planum supraseptale to the ventral margin of the interorbital septum/total length of the chondrocranium from the anterior tip of the nasal capsule to the most posterior margin of the auditory capsule, perpendicular to the sagittal plane
**11/13**	maximum length of the orbitotemporal region, in the midline, from the anterior and medial point of the planum supraseptale to the crista sellaris/maximum length of the chondrocranium from the most anterior point of the nasal capsule to the occipital condyle, parallel to the midline
**12/13**	maximum length of the otic-occipital region, in the midline, from the crista sellaris to the most posterior margin of the tectum/maximum length of the chondrocranium from the most anterior point of the nasal capsule to the occipital condyle, parallel to the midline
**4/13**	maximum width of the chondrocranium at the level of taeniae marginalis/maximum length of the chondrocranium from the most anterior point of the nasal capsule to the occipital condyle, parallel to the midline
**6/13**	maximum length of the planum supraseptale from its anterior and medial point to the most anterior point of the epioptic fenestra/maximum length of the chondrocranium from the most anterior point of the nasal capsule to the occipital condyle, parallel to the midline
**19/15**	maximum height of the parietotectal cartilage, perpendicular to its ventral edge/maximum length of the nasal capsule at the level of parietotectal cartilage from the tip of the nasal capsule to the aditus conchae (anterior part), parallel to the midline
**1/15**	maximum width of the nasal capsule at the level of parietotectal cartilage/maximum length of the nasal capsule at the level of parietotectal cartilage from the tip of the nasal capsule to the aditus conchae (anterior part), parallel to the midline
**20/16**	maximum height of paranasal cartilage, perpendicular to its ventral edge/maximum length of the nasal capsule at the level of paranasal cartilage from the aditus conchae (anterior part) to the lamina orbitonasalis, parallel to the midline
**2/16**	maximum width of the nasal capsule at the level of paranasal cartilage/maximum length of the nasal capsule at the level of paranasal cartilage from the aditus conchae (anterior part) to the lamina orbitonasalis, parallel to the midline
**2/17**	maximum width of the nasal capsule at the level of paranasal cartilage/total length of the nasal capsule from its anterior tip to the lamina orbitonasalis, parallel to the midline
**2/4**	maximum width of the nasal capsule at the level of paranasal cartilage/maximum width of the chondrocranium at the level of taeniae marginalis
**7/8**	maximum length of the epioptic fenestra/maximum width of the epioptic fenestra
**9/10**	maximum length of the optic fenestra/maximum width of the optic fenestra
**7/13**	maximum length of the epioptic fenestra/maximum length of the chondrocranium from the most anterior point of the nasal capsule to the occipital condyle, parallel to the midline
**9/13**	maximum length of the optic fenestra/maximum length of the chondrocranium from the most anterior point of the nasal capsule to the occipital condyle, parallel to the midline
**14/13**	maximum width of the otic-occipital region at the level of crista parotica/maximum length of the chondrocranium from the most anterior point of the nasal capsule to the occipital condyle, parallel to the midline
**3/4**	maximum width of the chondrocranium at the level of the planum supraseptale/maximum width of the chondrocranium at the level of taeniae marginalis
**5/6**	maximum length of the planum supraseptale from its anterior and medial point to the most anterior point of the optic fenestra/maximum length of the planum supraseptale from its anterior and medial point to the most anterior point of the epioptic fenestra
**22/21**	maximum height of epioptic fenestra/maximum height of the chondrocranium from the most dorsal margin of the planum supraseptale to the ventral margin of the interorbital septum
**23/21**	maximum height of optic fenestra/maximum height of the chondrocranium from the most dorsal margin of the planum supraseptale to the ventral margin of the interorbital septum
**22/24**	maximum height of epioptic fenestra/maximum length of the epioptic fenestra
**23/25**	maximum height of optic fenestra/maximum length of the optic fenestra
**24/18**	maximum length of the epioptic fenestra/total length of the chondrocranium from the anterior tip of the nasal capsule to the most posterior margin of the auditory capsule, perpendicular to the sagittal plane
**25/18**	maximum length of the optic fenestra/total length of the chondrocranium from the anterior tip of the nasal capsule to the most posterior margin of the auditory capsule, perpendicular to the sagittal plane

The proportions were analysed using PCA in PAST 4.03 to evaluate the morphological variation of the chondrocrania. PCA was performed on a correlation matrix derived from all proportions. For *Cr. palustris*, in which eight measurements from lateral view were missing (figure 3; table 1; electronic supplementary material, table S1), missing values were replaced by the average of the corresponding variables (*mean value imputation* method). Additional crocodilian specimens referenced in the literature were excluded from statistical analysis due to extensive missing data (figures 1–3; table 1), though their cranial morphology is qualitatively assessed and discussed later. This approach permitted us to quantify and compare the morphological diversity of mature chondrocrania across crocodilian species.

## 3. Results

### 3.1. Anatomical description of the chondrocranium of *Caiman crocodilus*

#### 3.1.1. The ethmoid region

**Figure 6.**
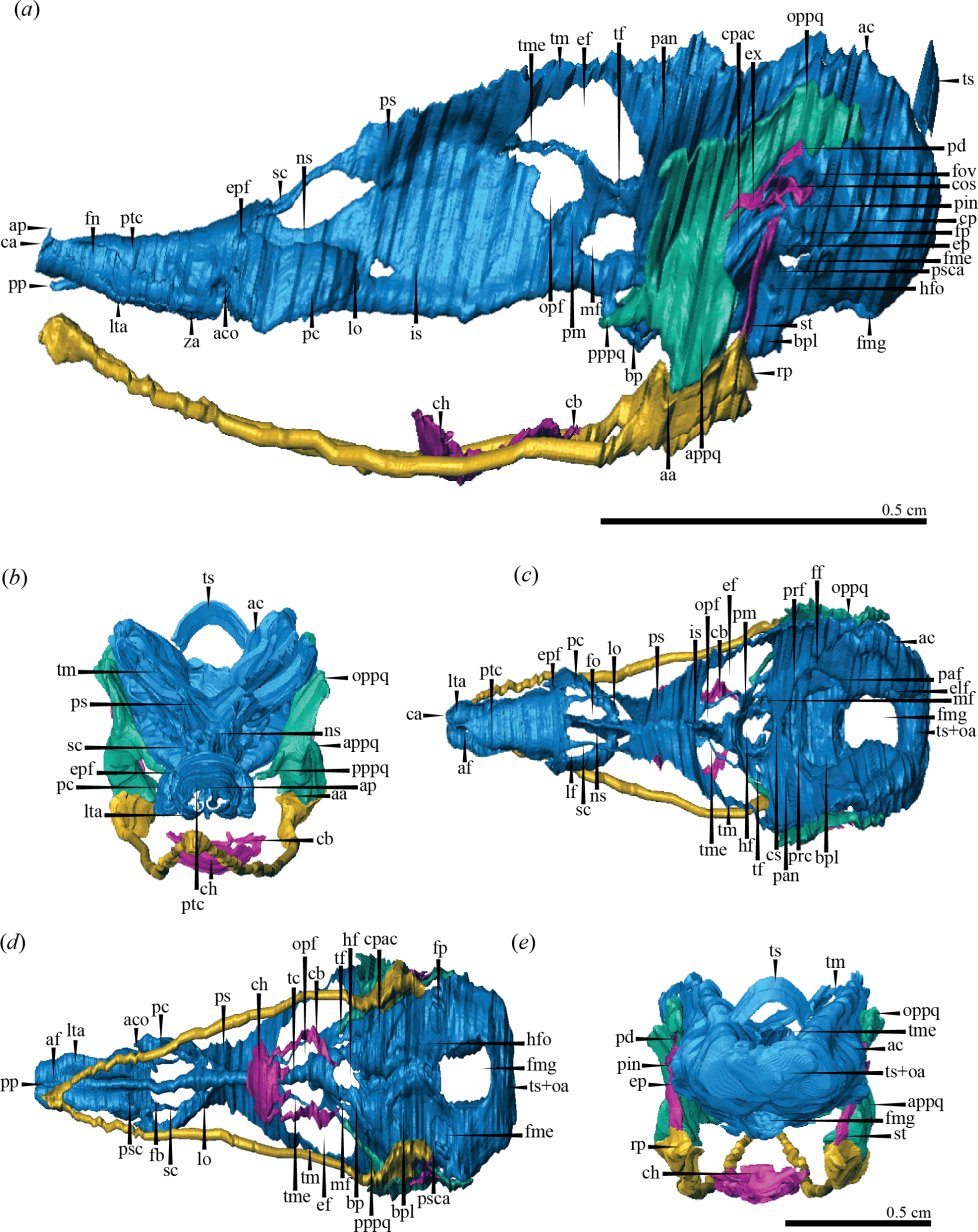
Three-dimensional model of the chondrocranium of *Caiman crocodilus*. Left lateral (*a*), anterior (*b*), dorsal (*c*), ventral (*d*) and occipital (*e*) views. Abbreviations: aa, articular area; ac, auditory capsule; aco, aditus conchae; af, apical foramen; ap, alar process; appq, articular portion of palatoquadrate; bp, basitrabecular process; bpl, basal plate; ca, cupola anterior; cb, cornu branchiale I; ch, corpus hyoidei; cos, columella shaft; cp, crista parotica; cpac, cochlear portion of the auditory capsule; cs, crista sellaris; ef, epioptic fenestra; elf, endolymphatic foramen; ep, epihyal; epf, epiphanial foramen; ex, extracolumella; fb, fenestra basalis; ff, facial foramen; fme, fissura metotica; fmg, foramen magnum; fn, fenestra narina; fo, fenestra olfactoria; fov, fenestra ovalis; fp, foramen perilymphaticum; hf, hypophysial fenestra; hfo, hypoglossal foramina; is, interorbital septum; lf, lateral fenestra; lo, lamina orbitonasalis; lta, lamina transversalis anterior; mf, metoptic fenestra; ns, nasal septum; opf, optic fenestra; oppq; otic process of palatoquadrate; paf, posterius acusticum foramen; pan, pila antotica; pc, paranasal cartilage; pd, processus dorsalis; pin, pars interhyalis; pm, pila metoptica; pp, prenasal process; pppq; pterygoid process of palatoquadrate; prc, prefacial commissure; prf, prootic fenestra; ps, planum supraseptale; psc, paraseptal cartilage; psca, processus subcapsularis; ptc, parietotectal cartilage; rp, retroarticular process; sc, sphenethmoid commissure; st, stylohyal; tc, trabecula communis; tf, trochlear foramen; tm, taenia marginalis; tme, taenia medialis; ts, tectum synoticum; ts + oa, tectum synoticum + occipital arches; za, zona annularis. Scale bar, 0.5 cm.

This region extends from the anterior to the posterior portion of the nasal capsules. It comprises both nasal capsules and their respective components, including the anterior cupolae, alar processes, parietotectal and paranasal cartilages, laminae transversalis anterior, prenasal process and lamina orbitonasalis, as well as the anterior segment of the nasal septum and the sphenethmoid commissures (figure 6*a*–*d*).

The nasal capsules are the most prominent paired structures in this region, positioned on either side of the nasal septum. Each nasal capsule consists of two distinct portions: an anterior, cone-like structure with the apex facing forward, and a posterior, globular structure that is laterally expanded. The anterior portion has a roof and lateral wall formed by the parietotectal cartilage, with a floor made by the lamina transversalis anterior (figure 6*a*–*c*). The parietotectal cartilage is a dorsolateral extension of the dorsal edge of the most anterior region of the nasal septum. As it projects laterally, it curves ventrally, but the lateral extension of this projection is not uniform along its entire anteroposterior length; it increases in length anteroposteriorly, until it reaches the zona annularis (figure 6*a*). The zona annularis is the region where the lateral wall of the nasal capsule (the parietotectal cartilage) meets the lamina transversalis anterior. The lamina transversalis anterior is a ventrally arched, anteroposteriorly elongated plate that extends from the ventral portion of the anterior nasal capsule wall (cupola anterior) to the zona annularis (figure 6*d*). The lateral edge of this lamina is free and dorsally oriented, while the medial edge is fused with the nasal septum along its entire length, except in the posterior section. Its posterior margin is curved. The posterior part of the medial border projects posteriorly and extends beyond the posterior edge of the lamina transversalis anterior, forming a small posterior projection known as paraseptal cartilage (figure 6*d*). Each lamina transversalis anterior is pierced anteromedially by a small, rounded foramen called the apical foramen, through which the ramus medialis nasi of the profundus branch of the trigeminal nerve exits (figure 6*c*,*d*). Medial to this foramen, the prenasal process—an odd, rounded, transverse structure—projects anteroventrally from the ventral border of the anterior portion of the nasal septum. The tip of the prenasal process curves dorsally and does not reach the level of the cupola anterior (figure 6*a*,*d*).

The cupola anterior forms the anterior wall of the nasal capsule and projects dorsally from the anterior region of the lamina transversalis anterior, extending to the level of the parietotectal cartilage (figure 6*a*). At this point, the dorsal and medial regions of the cupola anterior extend dorsally into a short alar process. A complete reconstruction of this area was not possible because some of the most anterior sections of the *Caiman crocodilus* embryo were missing. As a result, we could not fully observe the shape of the cupola anterior and the alar process. However, we were able to identify and describe some portions of these structures, confirming their presence in *Ca. crocodilus*. Moreover, our histological sections closely resemble those shown by Klembara [23] for *Alligator mississippiensis*, where both the cupola anterior and the alar process are also present.

The fenestra narina is a rather long and clearly visible opening in lateral and dorsal views (figure 6*a*,*c*). It extends from the cupola anterior (anterodorsally) to the zona annularis (posteroventrally), and is enclosed by the cupola anterior (anteriorly), the parietotectal cartilage (dorsally and posteriorly) and the lamina transversalis anterior (ventrally). In *Ca. crocodilus*, the fenestra narina is well developed, resulting in a small contact area between the floor and lateral wall of the nasal capsule. As a result, the zona annularis is considered poorly developed in this species.

The posterior portion of the nasal capsule shows a lateral wall formed by the paranasal cartilage and a posterior wall defined by the lamina orbitonasalis (figure 6*a*,*c*). There is no distinct floor or roof in this area. The paranasal cartilage is a robust structure located posterior to the parietotectal cartilage and ventral to the sphenethmoid commissures. Its height is similar to that of the parietotectal cartilage. In dorsal view, it has an approximate C-shape, with its posterior end medially continuous with the lateral border of the lamina orbitonasalis, which, in turn, is fused medially with the nasal septum (figure 6*c*). The medial section of the anterior border of the paranasal cartilage presents a short anterior projection. In ventral view, the wide fenestra basalis is delimited by the lamina transversalis anterior (anteriorly), the paranasal cartilage (laterally), the lamina orbitonasalis (posteriorly) and the nasal septum (medially) (figure 6*d*).

The boundary between the parietotectal and paranasal cartilages is defined not only by their distinct shapes but also by the presence of the concha nasalis and the epiphanial foramen (figure 6*a*–*d*). The concha nasalis is an invagination of the nasal capsule wall into the nasal cavity, containing a diverticulum of the nasal sac which is visible through the external opening known as aditus conchae (figure 6*a*,*d*). The epiphanial foramen, located above the aditus conchae, is a moderately sized opening through which the ramus lateralis nasi of the profundus branch of the trigeminal nerve exits, innervating the roof of the region (figure 6*a*–*c*). The dorsal margin of the epiphanial foramen forms a short projection that connects the parietotectal and paranasal cartilages.

The nasal septum is a vertical lamina situated along the midline, separating the two nasal capsules. It extends throughout the entire length of the ethmoid region, continuing into the orbitotemporal region where it transitions smoothly into the interorbital septum without a clear boundary (figure 6*a*,*c*). Its height (as well as the parietotectal cartilage in lateral view) gradually increases from its most anterior region until it reaches the area where the parietotectal cartilage meets the sphenethmoid commissures. At this point, its height decreases sharply before increasing again in a curved outline visible in lateral view, continuing into the orbitotemporal region as the interorbital septum (figure 6*a*). The thickness of the nasal septum also increases progressively from anterior to posterior and from dorsal to ventral (figure 6*c*,*d*). It is generally thicker ventrally, except for a sector near the middle of its anteroposterior length, i.e. at the level of the paraseptal cartilages and the posterior margin of the lamina transversalis anterior, where the ventral thickness is similar to the dorsal thickness.

The sphenethmoid commissures are paired, elongated structures that connect the ethmoid and orbitotemporal regions. They extend posterodorsally from the most posterior edge of the parietotectal cartilages to the anterior part of the planum supraseptale, running dorsally to the paranasal cartilages (figure 6*a*,*c*). In lateral view, the commissures are inclined, with the anterior portion positioned anteroventrally relative to the most posterior sector (figure 6*a*). In the dorsal view, two fenestrae are defined by the position of the sphenethmoid commissures: the medial fenestra olfactoria and the lateral fenestra (figure 6*c*). The fenestra olfactoria is delimited by the posterior border of the parietotectal cartilage (anteriorly), the sphenethmoid commissures (laterally), the anterior border of the planum supraseptale (posteriorly) and the dorsal margin of the nasal septum (ventromedially) (figure 6*c*). The lateral fenestra is the space between the sphenethmoid commissures (medially) and the dorsal border of the paranasal cartilage (laterally) (figure 6*c*).

#### 3.1.2. The orbitotemporal region

It is the middle area of the chondrocranium and lies between the ethmoid and otic-occipital regions. It extends from the planum supraseptale to the crista sellaris, encompassing the interorbital septum, trabeculae, trabecula communis, taeniae medialis and marginalis and the pilae metoptica and antotica (figure 6*a*,*c*,*d*).

The interorbital septum is the posterior extension of the nasal septum. Its height changes along its length from anterior to posterior: it first increases (continuing the same slope as the nasal septum), then slightly decreases and finally drops sharply in a vertical plane, where it contacts the pila metoptica, and ends in front of the hypophysial fenestra (figure 6*a*,*c*). The thickness of the septum is relatively consistent along its entire anteroposterior length, with a narrow cross section and a ventral edge that broadens into a teardrop shape (figure 6*c*,*d*). In lateral view, a large, irregularly shaped foramen (not a real opening, see §4) is visible in the most anterior and ventral part of the septum, just posterior to the lamina orbitonasalis (figure 6*a*). Most of the dorsal edge of the septum develops a dorsolateral projection on either side of the midline, called the planum supraseptale (figure 6*a*,*c*). The planum supraseptale is a thin, curved sheet of cartilage, roughly rectangular in shape and oriented anteroposteriorly. It connects anteriorly with the sphenethmoid commissure and posteriorly with the taenia medialis (medially) and taenia marginalis (laterally) (figure 6*a*,*c*). The taenia marginalis is a thin, elongated cartilaginous bar that attaches anteriorly to the posterior and lateral edges of the planum supraseptale and extends dorsolaterally backward, eventually contacting the anterior and dorsal surface of the otic capsule. The taenia marginalis passes dorsally to the pila antotica (figure 6*a*,*c*). The taenia medialis is a short, cylindrical structure that extends dorsolaterally backward from the posterior and medial edge of the planum supraseptale to the area where it broadens ventrally, continuing into the pila metoptica (figure 6*a*,*c*). The rectangular-shaped pila metoptica fuses posterodorsally with the anterior side of the ventral half of the pila antotica and ventrally with the dorsal edge of the most posterior part of the interorbital septum (figure 6*a*,*c*). At the junction of the pila metoptica and pila antotica, there is a foramen called the trochlear foramen, which is rounded on the left side and more anteroposteriorly elongated on the right side in our specimen (figure 6*c*). This foramen serves for the exit of the trochlear nerve [3,23]. Ventral to the trochlear foramen, there is the metoptic fenestra, a large opening delimited anteriorly by the pila metoptica, ventrally by the trabecula and posteriorly by the pila antotica (figure 6*a*,*c*). The oculomotor nerve and the arteria ophthalmica exit through this fenestra [23]. Two additional large openings are found in this region: the epioptic and optic fenestrae (figure 6*a*,*c*). The epioptic fenestra is positioned dorsal and lateral to the optic fenestra and is bounded by the planum supraseptale (anteriorly), taenia marginalis (dorsally), pila antotica (posteriorly), taenia medialis (anteroventrally) and pila metoptica (posteroventrally). The optic fenestra is enclosed by the planum supraseptale (anteriorly), taenia medialis (dorsally), pila metoptica (posteriorly) and interorbital septum (medially). The optic fenestra is about half the size of the epioptic fenestra, with its anterior border slightly more anterior than that of the epioptic fenestra. The optic nerve passes through the optic fenestra [23].

The trabecula communis dorsally gives rise to both the nasal and interorbital septa. In the described specimen, boundaries between these structures are no longer discernible (figure 6*a*,*c*,*d*). Initially, the trabeculae develop as two distinct elements along the midline of the ethmoid and orbitotemporal regions [2,14]. During ontogeny, these elements fuse to form the trabecula communis, which contributes dorsally to the formation of both the nasal and interorbital septa. In our *Caiman* embryo, the paired origin of the trabeculae [3,14] is evident only in the most posterior region of the trabecula communis, where branches remain separate in the posterior and ventral area of the orbitotemporal region (figure 6*d*). Each trabecula is a thick rod of cartilage diverging posterolaterally from the most posteroventral area of the trabecula communis toward the crista sellaris. The ventral surface of each trabecula presents a posteroventral projection, the basitrabecular process, which extends toward the ventral part of the cochlear portion of the otic capsule, though it does not make contact with it (figure 6*c*). This process is rounded in cross section at its most anterior end but it flattens dorsoventrally and widens laterally as it progresses posteriorly (infrapolar process; see Fernandez Blanco [24] and references therein). The trabeculae project posteroventrally and define the anterolateral boundary of the hypophysial fenestra, which is approximately triangular in shape and bordered posteriorly by the crista sellaris (figure 6*c*,*d*). The crista sellaris is a relatively long, tall, transverse cartilage bar (also known as acrochordal cartilage; see de Beer [2] and Yaryhin & Werneburg [14]) that forms the anterior margin of the basal plate (figure 6*c*). Its dorsal margin is free and extends dorsolaterally fusing with the pila antotica on each side. Ventral to this contact and dorsal to where it meets the trabecula, the crista sellaris is perforated by a small, rounded foramen that accommodates the abducens nerve.

The pila antotica is a robust, vertically oriented cartilage. Its ventral end tilts medially and connects with the dorsolateral edge of the crista sellaris, while its dorsolateral edge fuses with the ventral edge of the posterior section of the taenia marginalis (figure 6*a*,*c*). The posterior margin of the pila antotica forms the anterior border of the prootic fenestra, which is elliptical in shape, with a dorsoventrally elongated axis, and is bordered posteriorly by the anterior edge of the otic capsule. The ganglion of the trigeminal nerve is lodged within this fenestra [23].

#### 3.1.3. The otic-occipital region

This region extends from the area where the otic capsules attach to the floor (basal plate) by the prefacial commissures toward the posterior margin of the chondrocranium, where the tectum synoticum and occipital arches are located (figure 6*a,c–e*).

The otic capsules are large, paired structures located on either side of this region, each consisting of a vestibular and a cochlear component. The vestibular portion is prominent, globular in shape with an irregular contour, and positioned posterior to the cochlear portion, except for its most anterior part, which is located dorsally (figure 6*a*). The more elongated cochlear portion projects anteroventrally, from the anteroventral and lateral margin of the vestibular portion to the ventral surface of the basal plate, with which it fuses dorsally. The ventral portion of the cochlear portion lies dorsal to the basitrabecular process (figure 6*d*). The prefacial commissure connects the anteroventral margin of each capsule (vestibular portion) to the anterior and lateral area of the basal plate, and delimits the posterior margin of the prootic fenestra (figure 6*c*). The posterior margin of the dorsal part of this commissure, together with the anterodorsal edge of the cochlear portion, encloses the rounded facial foramen, providing the exit for the facialis nerve [23]. Just posterior to this foramen, in the anteroventral and medial wall of the otic capsule, two additional foramina are present: the anterius acusticum foramen and the posterius acusticum foramen. The anterius acusticum foramen is positioned dorsal and slightly anterior to the posterius acusticum foramen. A third, large foramen, the endolymphatic foramen, is located on the medial surface of the otic capsule, in the middle area of the posterior region of the otic capsule, dorsal to the acusticum foramina (figure 6*c*). The lateral wall of the otic capsule also contains two anteroposteriorly elongated foramina, situated anterior to the crista parotica: the dorsal fenestra ovalis and the ventral foramen perilymphaticum (figure 6*a*). The crista parotica, a laterally projecting prominence of the otic capsule, takes the shape of a vertically oriented ‘C’, opened anterior and laterally.

The floor of the otic-occipital region is formed by the basal plate (figure 6*d*). Its anterior margin is thickened and grows dorsally forming the crista sellaris, while its posterior edge defines the anterior boundary of the foramen magnum (figure 6*c*). The notochord runs along the midline of the basal plate, fully embedded within it. The processus subcapsularis projects dorsally and laterally from the middle region of the lateral edge of the basal plate, posterior to the cochlear portion and anterior to the hypoglossal foramina (see below). This tongue-shaped process extends laterally and below the otic capsule forming the floor of the most anterior part of the fissura metotica (see below) (figure 6*a*,*d*). Laterally, the processus subcapsularis overlaps the anterior half of the foramen perilymphaticum and the anterior sector of the fissura metotica (figure 6*a*,*d*). The fissura metotica is an opening delimited by the otic capsule and basal plate, extending from the posterior region of the cochlear portion to the hypoglossal foramina. The posterior part of this fissura serves for the exit of the glossopharyngeal, vagus and accessorius cranial nerves (IX, X and XI cranial nerves, respectively), as well as the vein jugularis [23]. The posterior and lateral region of the basal plate is perforated by three hypoglossal foramina arranged in an anteroposterior direction (figure 6*a*,*d*). The most anterior hypoglossal foramen is very small and is visible only in some slices. The remaining two foramina are rounded and of similar size. These hypoglossal foramina serve for the exit of the hypoglossal nerve (XII cranial nerve) [23]. The rhomboid-shaped foramen magnum is positioned along the midline in the posterior and ventral area of the otic-occipital region, medial to the otic capsules and posterior to the hypoglossal foramina (figure 6*d*,*e*). It is oriented obliquely, extending from anteroventral to posterodorsal, highlighting the flexure (i.e. trabeculopolar angle; see Fernandez Blanco [24]) among the two anterior chondrocranial regions and the otic-occipital region (figure 6*a*).

The otic capsules are medially connected in their posterior region by a cartilaginous band that is interpreted here as the tectum synoticum (figure 6*e*). The most anterior and dorsal portion of the tectum synoticum was identified as a separate element and likely corresponds to the anterior process of the tectum, similar to the configuration seen in other reptile species [33], including other *Caiman* species (see Fernandez Blanco [24]; figure 1*c*). Occipital arches can no longer be distinguished as separated elements and they are considered to be fused with the tectum [3].

#### 3.1.4. Mandibular arch

It consists of the Meckel’s cartilage and the palatoquadrate, present on each side of the skull (figure 6).

The palatoquadrate is a robust element positioned laterally to the pila antotica, the prootic fenestra and the anterior half of the otic capsule (figure 6*a*). It includes a main body (the articular portion), an otic process and a pterygoid process. The articular portion is a roughly rectangular plate, elongated dorsoventrally, with its largest surface positioned laterally to the neurocranium. It is located very close to the neurocranium but does not make contact with it. Its dorsal part projects posterodorsally, forming the otic process (figure 6*a*–*c,e*), while its ventral region expands lateromedially to form the articulation area with the Meckel’s cartilage (figure 6*a*,*b*,*e*). From the medial and anterior side of this ventral region, the pterygoid process of the palatoquadrate extends medially and anteroventrally (figure 6*a*,*b*,*d*). This process, roughly circular in cross section, tapers along its length and terminates in a free end at the same level as the trabeculae, positioned laterally to them.

The Meckel’s cartilage is a long, slender rod oriented anteroposteriorly. Its cylindrical cross section maintains a consistent diameter throughout most of its length, except at the most anterior and posterior ends. Anteriorly, it broadens dorsoventrally and meets its counterpart at the midline (figure 6*a*). Posteriorly, the Meckel’s cartilage expands dorsoventrally, and its dorsal surface broadens mediolaterally forming the rectangular-shaped articular area where it connects with the ventral region of the palatoquadrate’s articular portion. In the posteromedially region of this articular area, a short, steeply vertical retroarticular process projects posteroventrally and slightly medially (figure 6*a*,*b*,*e*).

#### 3.1.5. Hyoid arch and branchial arches

The dorsal portion of the hyoid arch forms the columella auris, the pars interhyalis and the epihyal and stylohyal cartilages. The columella auris is a complex structure comprising the footplate, columella shaft, extracolumella and dorsal process [34]. The ventral portion of the hyoid arch, along with the branchial arches, forms the hyobranchial apparatus (also referred to as the hyoid apparatus; see Schumacher [35] and Fernandez Blanco [24]) (figure 6).

The columella auris is an elongated structure oriented along a lateromedial axis (the columella shaft), with expanded ends (figure 6*a*). Laterally, it continues as the extracolumella, positioned anteriorly and ventrally, while medially, the footplate of the columella rests against the fenestra ovalis (otic capsule), leading to the inner ear. The extracolumella extends laterally to connect with the tympanic membrane. A long process (processus dorsalis) projects posterodorsally from the dorsal area of the columella shaft, medially to the extracolumella (figure 6*a*,*e*). The posteroventral area of the extracolumella contacts the pars interhyalis, a crescent-shaped structure projecting posteriorly. The pars interhyalis is continuous ventrally with the epihyal cartilage which, in turn, is continuous ventrally with the stylohyal cartilage (figure 6*a*,*e*). The stylohyal cartilage articulates with the mandible at the region where the articular area of the Meckel’s cartilage meets the retroarticular process. Both the epihyal and stylohyal cartilages are slender and elongated, linking the columella auris complex to the mandible.

The hyobranchial apparatus consists of the corpus hyoidei and a pair of cornu branchiale I, positioned along the midline, ventral to the orbitotemporal region of the neurocranium (figure 6*a*–*e*). The corpus hyoidei is a warped, trapezoidal plate with an irregular outline and a concave dorsal surface that accommodates the larynx and the first tracheal rings. The anterior edge of the corpus hyoidei presents one middle and two lateral processes, separated by two large indentations or notches (figure 6*d*,*e*). While each of these processes has a relatively straight edge, their arrangement imparts a slight curvature to the whole anterior edge of the corpus hyoidei. Posterior to the middle anterior process, the corpus hyoidei exhibits a rounded thinning in the cartilage (concave-like), likely indicating recent cartilage formation in this area (see Fernandez Blanco [24]) (figure 6*e*). Posterior to each anterior lateral process, a cornu branchiale I is articulated. Each cornu branchiale I is a long, cylindrical cartilage that tapers and curves medially at its distal end (figure 6*d*). The corpus hyoidei gradually narrows posteriorly from its widest area, at the level of the anterior lateral processes, and terminates in two posterior lateral processes. These posterior processes are elongated and slender compared to the anterior lateral processes, and they are separated by a middle posterior notch at the posterior edge of the corpus hyoidei (figure 6*d*).

### 3.2. Principal component analysis

**Figure 7.**
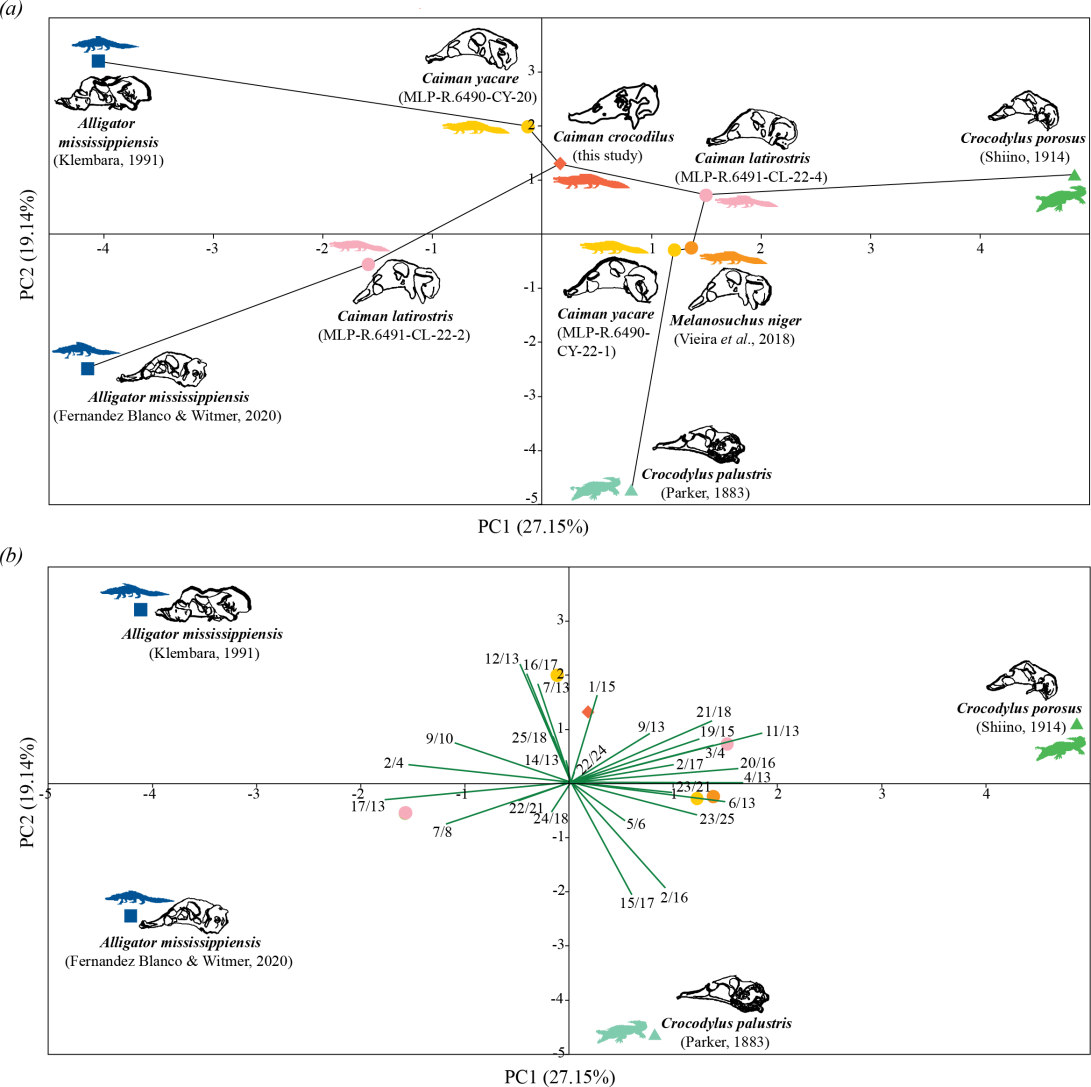
Principal component analysis. (*a*) PCA scatterplot with PC1 on the *X*-axis and PC2 on the *Y*-axis, showing morphological variation of the chondrocranial proportions and minimal span tree. (*b*) Biplot projecting chondrocranial proportions on the scatterplot with PC1 versus PC2.

The PCA resulted in nine principal components explaining the total accumulated variance. Only the first three PCs were considered since they showed a clear separation among species. PC1 accounts for 27.15% of the total morphological variation, whereas PC2 explains 19.14% of it (figure 7*a*; electronic supplementary material, table S2). PC3 explains 16.68% (electronic supplementary material, figure S1a and table S2) and, together with the first two PCs, accounts for nearly 63% of the accumulated variance. We plotted PC1/PC2 and PC1/PC3 since PC1 shows the highest percentage of explained variance and morphological signal.

In the scatterplot of PC1 versus PC2, crocodilian species are broadly distributed along the four quadrants, and the three groups (Alligatorinae, Caimaninae, Crocodylinae) are relatively well separated from each other along PC1 (figure 7*a*; electronic supplementary material, table S3). Alligatorids (Alligatorinae + Caimaninae) are located from negative values of this component to positive ones (from −4.14 to 1.49), while crocodylids (Crocodylinae) are positioned only in the positive values of PC1 (from 0.81 to 4.86). Alligatorines (*A. mississippiensis*) are well separated from caimanines (*Caiman* and *Melanosuchus*) along this PC. The two *A. mississippiensis* specimens are in the extreme negative values of this component (around −4.04 and −4.14) whereas caimanines are restricted to values around the centre of the diagram (from −1.58 to 1.49). Regarding PC2, most specimens (except for *Crocodylus palustris*) are found in the area delimited by 3.19 and −2.47, which correspond to the position of the two *A. mississippiensis* specimens. The two alligatorines are quite distant from each other, particularly along PC2. A similar pattern is observed for *Crocodylus porosus* and *Cr. palustris* but, in this case, both PC1 and PC2 contribute significantly to their separation. Along PC2, *Cr. palustris* remains far from the rest of the sample (around −4.72), and *Cr. porosus* is closer to caimanines (around 1.09) than to *Cr. palustris*. Despite this, both crocodylines are well apart from caimanines, and *Cr. palustris* is even closer to caimanines than *Cr. porosus* (see minimal span tree in figure 7*a*). *Caiman yacare* (MLP-R.6490-CY-22-1) is the closest specimen to *Cr. palustris*, and *Ca. latirostris* (MLP-R.6491-CL-22-4) is the closest specimen to *Cr. porosus*. Concerning caimanines, most of them are restricted to the central region of the scatterplot (positive values of PC1 and/or PC2), closer to each other and well separated from alligatorines and crocodylines. Specimens of the same species (*Ca. latirostris* and *Ca. yacare*, separately) are closer to specimens of the other species. Thus, the *Ca. latirostris* (MLP-R.6491-CL-22-4) specimen is closer to the *Ca. yacare* (MLP-R.6490-CY-22-1) specimen and also to *Melanosuchus niger* than to the other specimen of *Ca. latirostris*. The same pattern is observed for *Ca. yacare*: the *Ca. yacare* (MLP-R.6490-CY-22-1) specimen is closer to *Ca. latirostris* (MLP-R.6491-CL-22-4) and to *Me. niger* than to the other specimen of *Ca. yacare*. Regarding *Ca. crocodilus*, it is nested within caimanines, particularly close to the *Ca. yacare* (MLP-R.6490-CY-20) specimen (figure 7*a*). *Melanosuchus niger* is closer to the *Ca. yacare* (MLP-R.6490-CY-22-1) specimen than to the rest of the sample.

In the scatterplot of PC1 versus PC3, the species distribution is quite similar to that of the PC1/PC2 biplot (electronic supplementary material, figure S1a and table S3). Alligatorines, caimanines and crocodylines are still fairly well separated along PC1 and, within each group, specimens from the same species are still distant from each other. Compared to the PC1/PC2 biplot, the two specimens of *A. mississippiensis* swapped their position along PC3, and the *A. mississippiensis* [23] specimen is now in the negative values of this component (−3.40) whereas the other *Alligator* specimen is in the positive ones (1.10). Similarly, *Cr. porosus* and *Cr. palustris* switched their position; *Cr. palustris* is closer toward the zero value of PC3 (−1.55) and *Cr. porosus* is in the negative values of PC3 (−2.77). Therefore, *Cr. palustris* is relatively closer to caimanines, particularly near to *Me. niger*, while *Cr. porosus* is far away from all of them and from alligatorines (electronic supplementary material, figure S1a). Regarding caimanines, most of them are within the double positive quadrant or very close to it, except for the *Ca. latirostris* (MLP-R.6491-CL-22-2) specimen which is somewhat separated from it. As in the PC1/PC2 diagram, the two specimens of *Ca. latirostris* are well distant from each other and are closer to the *Ca. yacare* specimens. Likewise, the *Ca. yacare* (MLP-R.6490-CY-22-1) specimen is closer to the *Ca. latirostris* (MLP-R.6491-CL-22-4) specimen, *Me. niger* and *Ca. crocodilus* than to the other *Ca. yacare* specimen.

Concerning anatomical proportions that mostly explain chondrocranial variation, we obtained the following results. Regarding the PC1 loadings of each variable, 11 variables were negatively correlated with it, with two of them having the most negative contributions: 17/13 (−0.322) and 2/4 (−0.281). The remaining 16 variables were positively correlated, with the highest 11/13, 4/13 and 20/16 (0.331 to 0.290) (figure 7*b*; electronic supplementary material, table S4). Positive values of PC1 are related mainly to skulls with longer and wider orbitotemporal regions and taller nasal capsules (paranasal cartilage), whereas negative values are associated mostly with larger and wider nasal capsules. Regarding the three proportions that most contribute to the positive values of the PC1, specimens with high values of these proportions are towards the right side of the diagram while low values are positioned towards the left side. Thus, *Cr. porosus* presents the highest values for those proportions presenting the largest influence of the length and width of the orbitotemporal region and the height of the paranasal cartilage in its morphology. This means that *Cr. porosus* presents the largest and widest orbitotemporal region and the taller paranasal cartilage. Specimens with low values for these proportions (towards the left side of the diagram) have short, narrow orbitotemporal regions and low paranasal cartilages. The two *A. mississippiensis* specimens represent this extreme having the shortest and narrowest orbitotemporal regions and the lowest paranasal cartilage. Besides, *Cr. porosus* presents a paranasal cartilage taller than long, while the other crocodilian specimens present the opposite condition (longer than taller). Regarding the two proportions that most contribute to the negative values of PC1, there is a gradient on the disposition of the specimens from left to right in this component. The highest values of these proportions are towards the negative values of PC1 and are represented by both specimens of *A. mississippiensis* that present the largest nasal capsule and the widest paranasal cartilage. The lowest values for the length of the nasal capsule and the width of the paranasal cartilage belong to *Ca. yacare* (MLP-R.6490-CY-22-1) and *Cr. porosus*. Therefore, *Ca. yacare* (MLP-R.6490-CY-22-1) and *Cr. porosus* have the shortest nasal capsule and the narrowest paranasal cartilage.

Regarding the PC2 loadings of each variable, eleven variables were negatively correlated with it, of which two had the most negative contributions: 15/17 (−0.373) and 2/16 (−0.351). The remaining 16 variables were positively correlated, with the highest being 16/17 (0.395) and 12/13 (0.362) (figure 7*b*; electronic supplementary material, table S4). Positive values of PC2 are related mainly to skulls with more elongated paranasal cartilages and otic-occipital regions whereas negative values are mostly associated with skulls with more elongated parietotectal and wider paranasal cartilages. Regarding the two proportions that most contribute to the positive values of PC2, specimens with high values of these proportions are positioned on top of the plot while low values are towards the bottom. Thus, *A. mississippiensis* [23] presents the highest values for those proportions, having the skull with the most elongated paranasal cartilages and otic-occipital region. Specimens with low values for these proportions (towards the bottom) present the opposite morphological condition and *Cr. palustris* represents this extreme. Regarding the two proportions that most contribute to the negative values of PC2, there is a gradient on the disposition of the specimens from bottom to top in the plot. *Crocodylus palustris* has the highest (most negative) values for these proportions and the associated morphological traits (most elongated parietotectal cartilages and the widest paranasal cartilages), while *Ca. yacare* (MLP-R.6490-CY-20) and *A. mississippiensis* [23] present the lowest values, showing the opposite associated morphological condition. In all chondrocrania, the width of the paranasal cartilage is larger than its length.

For PC3 loadings of each variable, 10 variables were negatively correlated with it, of which only one had the most negative contribution: 9/13 (−0.317). The remaining 17 variables were positively correlated, with the highest being 14/13 (0.436), 22/21 (0.325) and 22/24 (0.323) (electronic supplementary material, figure S1b and table S4). Positive values of PC3 are associated mostly with skulls with wider otic-occipital regions and taller epioptic fenestrae while negative values are related mainly to skulls with longer optic fenestra. Regarding the three proportions that most contribute to the positive values of PC3, specimens with high values of these proportions are positioned on top of the plot while low values are towards the bottom. Thus, both specimens of *Ca. yacare* present the highest values for those proportions, having the widest otic-occipital regions and the tallest epioptic fenestrae. Specimens with low values for these proportions (towards the bottom) present the opposite morphological condition, and *Cr. palustris* and *Ca. latirostris* (MLP-R.6491-CL-22-2) represent this extreme. Regarding the proportion that most contributes to the negative values of PC3, there is a gradient on the disposition of the specimens from the lower to the upper part of the plot. *Crocodylus porosus* has the highest (most negative) value for this proportion and the associated morphological trait (the longest optic fenestra), while *Ca. yacare* (MLP-R.6490-CY-20) presents the lowest value, showing the opposite associated morphological condition.

### 3.3. Impact of missing data and estimation methods on principal component analysis

Limitations in data completeness and specimen availability within tempus optimum have affected the scope of our morphometric analyses. Among these, the chondrocrania of *Mecistops cataphractus* and *Crocodylus niloticus* are documented in the literature, but their inclusion in our morphometric analyses was precluded by the lack of sufficient quantitative data, as these records are based on sagittal and paramedian sections only (*Cr. niloticus* [3; fig. 85a], *Me. cataphractus* [32; fig. 26c]) (figure 4; table 1). Similarly, incomplete datasets for certain specimens (*A. mississippiensis* [27; plate 65, fig. 8], *Cr. porosus* [5; fig. 1b], *Me. niger* [26; fig. 3a]) limited their inclusion into the present study (figures 1–3; table 1). On the other hand, there are additional specimens described in the literature, but as they were not within the tempus optimum [5], they were not included in our analyses (*A. mississippiensis* [23; plates 6, 8, 9], *Cr. palustris* [27; plate 69, fig. 8], *Cr. porosus* [31; fig. 1] and *Me. cataphractus* [32; figs 18, 19 and 21]) (figures 1, 3 and 4; table 1).

Regarding estimated measurements in *Cr. palustris*, when this species is excluded from the analysis, the overall species distribution in the morphospace remains largely consistent, with only minor shifts in specimen positions (electronic supplementary material, figure S2). Even in this scenario, only minor positional shifts occurred, and the separation among alligatorines, caimanines and crocodylines is preserved, with *Cr. porosus* still positioned far from other species. To further validate these findings, supplementary analyses using an alternative estimation method (e.g. iterative imputation) confirmed the overall robustness of our findings, with clear separation between big clades and consistent clustering patterns, although in this case, the differentiation occurred along PC2 (electronic supplementary material, figure S3). Notably, in this case, *Cr. palustris* is positioned farther from all other specimens, including *Cr. porosus*.

## 4. Discussion

Using histological sections and digital tools of embryonic tissues, we reconstructed the chondrocranium of *Caiman crocodilus* and identified unique chondrocranial features that may have systematic significance. We have detected an epiphanial foramen, for the passage of the lateral ethmoidal nerve in *Ca. crocodilus*, that is absent in *Crocodylus* [2,3,23] but present in *Alligator mississippiensis* [3,23], *Caiman latirostris* [24] and *Caiman yacare* [24]. Its presence in these species supports the assertion of Fernandez Blanco [24] that the epiphanial foramen is a distinctive feature of Alligatoridae [23,24]. Similarly, the alar process could also serve as a diagnostic trait for Alligatoridae. It appears in *Ca. crocodilus* (our three-dimensional model), *Ca. latirostris* [24], *Ca. yacare* [24] and *A. mississippiensis* [23,27], but is absent in *Crocodylus* [2,3]. Vieira *et al.* [26] did not report an epiphanial foramen or an alar process in *Melanosuchus*, though the apparent lack of these structures is likely a bias of the methodology applied by this author, as the clearing and staining technique can sometimes obscure certain tiny features [36]. However, even though the alar process was not mentioned by this author, it can be identified in the lateral view of the chondrocranium of *Melanosuchus* (see Vieira *et al.* [26, fig. 3d]). Additionally, in contrast to Crocodylinae, all specimens of Alligatoridae showed consistently elongated sphenethmoid commissures [23,24,26]. While the exact developmental origin of these structures—whether ethmoid or orbitotemporal—remains unclear, it is a reliable trait for identifying large clades. The morphological variation of the sphenethmoid commissures, including their presence, absence or differential development, has also been observed in other reptiles and even within the same taxonomic group (e.g. [37–39]).

Regarding the relationship between the neurocranium and splachnocranium, the nature of the connection between the otic process of the palatoquadrate with the otic capsule and the dorsal process of the columella auris may also have systematic significance. In *Crocodylus porosus*, these connections are formed through connective tissue [2,3] or cartilaginous fusion [2,40], whereas in *Ca. crocodilus*, these structures remain separate with a simple articulation between them. This last condition is also found in *Ca. latirostris* and *Ca. yacare* [24] and might be unique for *Caiman*. In turn, there is no ascending process of the pterygoid process of the palatoquadrate in *Ca. crocodilus* as well as in *Ca. latirostris* and *Ca. yacare* [24], while it was identified in *Cr. porosus* [2]. Thus, this might be an additional distinguishing trait of *Caiman*, similar to other reptile taxa such as pleurodire turtles [30].

In relation to visceral arches, we discovered a recess in the anterior and medial portion of the corpus hyoidei of *Ca. crocodilus* that reminds one of the typical foramina in the corpus hyoidei of *Ca. yacare* and *Ca. latirostris* [24]. We argue that this concavity may have originally developed as a complete foramen which began to obliterate during further development. This is why it is detected as a thin circular area in our three-dimensional model at the same position where foramina were observed in *Ca. yacare* and *Ca. latirostris* by Fernandez Blanco [24]. Following this idea, the presence of these foramina could be temporary, occurring only in a particular period of the embryonic development of *Caiman* species. In this sense, this feature is noteworthy not only because it is distinctive of the genus, but also because it has not been previously reported in the literature in other crocodilian or reptile species. It would be valuable to investigate the developmental basis of this morphological trait and assess whether its potential ontogenetic variation carries functional implications, particularly given that this region serves as the attachment site for muscles involved in tongue movement.

On the other hand, no notable morphological differences were identified among *Ca. crocodilus*, *Ca. yacare* and *Ca. latirostris*, as the chondrocranial structure of *Ca. crocodilus* closely corresponds to anatomical descriptions of the other *Caiman* species (see Fernandez Blanco [24]). We did identify a relatively large foramen in the anteroventral region of the interorbital septum that may represent a distinctive feature of *Ca. crocodilus*, but our findings are preliminary and a larger sample size is necessary before drawing broader conclusions. In fact, upon closer examination, a detailed analysis of histological sections reveals that is not a true opening, but rather a localized and extreme thinning of the wall, where distinguishing chondrocytes and defining precise boundaries become challenging (electronic supplementary material, figure S4). Something similar was described by Yaryhin & Werneburg [14] as fenestra septalis in *Lacerta*, and it appears to be filled with a thin membrane of cartilaginous matrix, rather than being a true fenestra. Notably, such a feature has not been documented in crocodilians thus far (because no histological sections were studied in this region). Bellairs & Kamal [3] described the interorbital septum of crocodilians as typically imperforate, while Vieira *et al.* [26] noted an unstained area in this region of *Melanosuchus* embryos, suggesting incomplete formation rather than a fully developed structure. We propose that the presence of this non-true foramen, or fenestra septalis, in *Ca. crocodilus* may result from a delayed chondrification similar to that observed in *Melanosuchus*, rather than representing a species-specific characteristic.

### 4.1. Quantitative analyses

Our results suggest that chondrocranial proportions are a reliable taxonomic indicator, clustering species according to phylogenetic relationships in morphospace. Despite the limited sample size, we observed a taxonomic grouping that is consistent with our initial hypothesis that evolutionary proximity among crocodilians is mirrored in the early cranial cartilage anatomy, and we expect that increasing the sample size in upcoming studies will yield more accurate results. Closely related species showed greater morphological similarity along PC axes, as evidenced by the separation of Alligatoridae and Crocodylidae along PC1 (figure 7*a*; electronic supplementary material, figure S1a), although some minor overlap was observed between *Crocodylus palustris* and Caimaninae. Within Alligatoridae, the clades Alligatorinae and Caimaninae also occupy distinct regions of the morphospace, reflecting their phylogenetic distance and further highlighting the method’s ability to differentiate between phylogenetically closely related groups. It can be seen that the two *Crocodylus* specimens and the *A. mississippiensis* individuals are positioned relatively far from one another, while caimanine specimens tend to cluster more closely together (figure 7*a*). This pattern in the morphospace may reflect species-specific ranges of morphological variation. Namely, the genus *Crocodylus* might exhibit a broad spectrum of morphological diversity, explaining the observed distance between *Cr. palustris* and *Cr. porosus* and the minor overlapping with caimanines (figure 7*a*; electronic supplementary material, figure S1a). On the other hand, morphological differences between the two *A. mississippiensis* specimens reflected in our dataset may be related to a slightly different development timing, with one specimen displaying features of a more advanced stage when compared to the other. Both specimens were initially included in our study because they exhibited a fully formed chondrocranium, as noted in their original descriptions [23,28]. However, a closer and *a posteriori* examination revealed developmental distinctions. In lateral view, the *A. mississippiensis* specimen described by Fernandez Blanco & Witmer [28] exhibits a well-defined trabeculopolar angle, whereas this feature seems to be absent in the other *A. mississippiensis* specimen. Additionally, the otic capsule in the specimen from Fernandez Blanco & Witmer [28] shows a posteroventral inclination, further highlighting this angle, while the otic capsule is aligned with the rest of the chondrocranium in the specimen from Klembara [23]. The trabeculopolar angle is a transient feature, prominent in early stages of development and disappears quickly during embryonic ontogeny in *Caiman* species [24]. As such, the absence of this angle and a fully straightened chondrocranial axis (including otic capsules) in the specimen of *A. mississippiensis* from Klembara [23] suggests it belongs to a slightly later developmental stage than the specimen of *A. mississippiensis* from Fernandez Blanco & Witmer [28]. These developmental differences would give rise to morphological variation in the cartilages of the nasal and otic capsules, influencing chondrocranial proportions and contributing to the separation of these specimens along PC2 and PC3 in the morphospace (figure 7*a*; electronic supplementary material, figure S1a). Furthermore, the dorsal views of these two *Alligator* specimens (to the naked eye) reveal differences in both shape and size of the optic and epioptic fenestrae that were not captured by the PCA. In this regard, a few measurements of the fenestrae (length, width and height) were slightly significant in PC3, contributing to the explanation of morphological changes and species differentiation. In relation to the fenestrae, we noticed a pronounced size difference in *Ca. crocodilus*, where the optic fenestra was about half the size of the epioptic fenestra, and its anterior border extended slightly further forward than the epioptic fenestra. Although this difference in the anterior extension could be an artefact of the reconstruction method, this feature has already been identified in *Caiman* by Fernandez Blanco [24], showing ontogenetic variation. Future research addressing this type of morphological and ontogenetic variation and its morphofunctional implications would be particularly important as this is the area where the optic nerve passes and the eyeball is located. These observations emphasize the subtle yet significant role of developmental timing in shaping chondrocranial morphology and its representation in the morphospace. Increasing the number of samples, studying different developmental stages and conducting more detailed analyses using two- or three-dimensional geometric morphometrics would be beneficial in the future to obtain more precision and more robust results.

### 4.2. Data limitations and methodological considerations

This study contributes to the limited dataset of fully reconstructed crocodilian chondrocrania, increasing the number of species with complete data to about one-third of the extant morphological diversity. Despite some sample limitations (e.g. incomplete views, missing measurements or specimens that do not fall within the tempus optimum stage) may have introduced small biases, influencing the positions of specimens in the morphospace (particularly true for *Cr. palustris*, *A. mississippiensis* and *Melanosuchus niger*), our tests indicate that the overall structure of the morphospace is robust. The use of different imputation methods, as well as the exclusion of *Cr. palustris* from the analyses, yielded consistent results: individual species preserved their relative positions and major clades remained clearly separated. Besides, the extrapolation of measurements from dorsal to ventral views may have influenced the morphospace distribution of species, particularly for the specimen of *A. mississippiensis* from Fernandez Blanco & Witmer [28] and for *Melanosuchus*. However, previous studies facing similar challenges used comparable methods and achieved equally valid results [41]. Therefore, we consider that the inclusion of estimated or extrapolated values has only minor influence on the observed morphological patterns and value approximation has minimal effects on morphospace distribution. Nonetheless, we emphasize the importance of increasing both the number of specimens and species sampled, as well as improving data completeness in future studies. This will allow for more precise shape quantification and reduce the reliance on approximations, thereby strengthening the statistical power of comparative analyses across Crocodylia.

### 4.3. Morphofunctional and ecological drivers of chondrocranial variation in crocodilians

The three crocodilian groups (Alligatorinae, Caimaninae and Crocodylinae) were differentiated mainly by the proportions of the snout and the orbitotemporal region. Morphological extremes were represented by *A. mississippiensis* (i.e. Alligatorinae), where the nasal region plays a dominant role in shaping morphology, and *Cr. porosus* (i.e. Crocodylinae), where the orbitotemporal region emerges as the most influential feature. Morphological distinctions between Crocodylinae and Alligatorinae chondrocrania may reflect a combination of ecological, geographical and evolutionary factors that could have shaped specific adaptations in each lineage. The larger influence of the nasal capsule in *A. mississippiensis* may be linked to its habitat and ecological demands. This species is nowadays distributed across the southeastern United States, typically inhabiting lakes, swamps, wetlands and vegetated areas (e.g. [42–46]). In these environments, shoreline vegetation plays a critical role in providing nest material for this species [46–49], supplying protection for hatchlings [46,50] and supporting hunting activities [46]. In turn, densely vegetated surroundings limit visibility. Facing these conditions, enhanced olfactory sensitivity and respiratory adaptations may offer significant advantages in this type of habitat. A more developed nasal capsule would increase the surface area available for the olfactory epithelium and the olfactory bulb, improving prey detection in enclosed aquatic environments and optimizing airflow in low-oxygen conditions such as marshy areas. A positive relationship between larger nasal cavities and improved olfactory function has been suggested in other groups such as turtles, birds and mammals (e.g. [51–56]), and a larger olfactory bulb has also been associated with a higher olfactory sense (e.g. [51,53–55,57–60]). Additionally, the size of the nasal cavities has been linked to diverse factors such as habitat occupation, thermoregulation and sound production (e.g. [54–56,61,62]). In particular, some researchers claim that although all crocodilians are vocal species, *A. mississippiensis* may have developed the most advanced vocalization capacity as an adaptation to its dense vegetated habitat, where visual communication is limited [63–65]. Eventually, in future studies, it would be valuable to assess the evolutionary implications of the potential morphofunctional relationship between nasal capsule morphology and vocalization ability in crocodilians. On the other hand, the prominence of the orbitotemporal region in *Cr. porosus* is likely related to its habitat and feeding ecology, particularly in the more open and fluctuating environments this species occupies [44,66,67]. *Crocodylus porosus* is the largest crocodilian species with an opportunistic diet, and one of the most extensive geographic ranges, demonstrating adaptability to both freshwater and marine environments (e.g. [67–72]). Its large body size [67,70,72] provides an extensive surface area for muscle attachment and the development of great muscle volume, facilitating the development of strong jaw muscles capable of generating high forces [35,73]. It has been proposed that bite forces in crocodilians are controlled by body size [21,74]. So, this anatomical feature and a more prominent orbitotemporal region likely support the presence of strong jaw adductor muscles, essential for capturing larger or hard-bodied prey items. The bigger the body size of *Crocodylus*, the bigger the prey size they can obtain. Consequently, the broad geographic distribution of *Cr. porosus*, spanning diverse environments, likely favoured orbitotemporal adaptations that enhance mechanical strength, enabling it to capture a wide variety of prey with different sizes and hardness. In contrast, the more restricted range of *A. mississippiensis*, confined to North America (e.g. [43–46]), may have driven selective pressures favouring nasal adaptations suited to specific and restricted vegetated habitats. Supporting this idea, Lewis *et al*. [45] argued that the home range and habitat selection of a species are constrained by both biotic and abiotic parameters. The idea that the final shape of the chondrocranium, along with variations in the timing of chondrification across different skull regions, may reflect species-specific feeding and ecological specializations has been previously proposed by other authors (e.g. [14,30,41]). However, this hypothesis still requires evaluation within a broader phylogenetic context to provide deeper insights into the morphofunctional integration of the skull. In this sense, as the functional morphology of the chondrocranium remains poorly understood, a detailed analysis of head and neck muscle attachments to the chondrocranium during early skull development could enhance our understanding of the functional factors shaping chondrocranial diversity.

Our findings also show that chondrocranial morphology reflects early taxonomic differentiation, as our sampling corresponds to developmental stages prior to, or just at, the onset of ossification, when the first cranial bones become visible (i.e. tempus optimum [5]). Given the close developmental association between cartilage and bone, anatomical changes in cartilaginous structures are likely to influence subsequent osteocranial development and morphology, as has been evidenced in other vertebrate taxa [8,9,11,12]. Indeed, early developmental modifications can have a significant impact on adult phenotype [21,75]. Therefore, interspecific differences detected in the chondrocranium during early embryogenesis are expected to persist, at least in part, throughout cranial ossification. Specifically, we consider that the morphological and taxonomic patterns detected at these early stages may anticipate similar distinctions in the ossified skull. Nonetheless, while our results reveal clear morphological distinction among the three crocodilian clades, a geometric morphometric analysis of ossified embryonic skulls showed a contrasting pattern. Morris *et al*. [21,76] found that embryonic skulls of extant crocodilians—already undergoing ossification—exhibit a unique and indistinguishable shape and cluster within a conserved region of morphospace, with significant anatomical changes just until before hatching. This pattern of morphological similarity occurs when cranial bones are already well developed (see Morris *et al.* [21]), which corresponds to later stages than those examined in our contribution. One possible explanation for the contrasting pattern between cartilaginous and ossified skulls is that the embryos analysed by Morris *et al.* [21] may display chondrocranial morphotypes already influenced by the onset of ossification, moving them beyond the tempus optimum [5]. Ultimately, these data suggest that, while the chondrocranium captures early, taxonomically informative morphological variation, such distinctions may become increasingly obscured as ossification progresses, further emphasizing the critical importance of developmental timing in comparative morphological analyses.

## 5. Conclusions

This study provides the first complete three-dimensional reconstruction of the chondrocranium of *Caiman crocodilus* based on histological sections and digital modelling. This is significant given the scarce data on embryonic cranial anatomy in extant crocodilians. Our results reveal a clear phylogenetic signal, with species clustering according to big crocodilian clades, and support early developmental differentiation. *Caiman crocodilus* showed strong morphological affinity with other *Caiman* species, and certain features—such as the fenestra septalis—likely reflect developmental variation rather than species-specific traits. We also identified diagnostic characters for *Caiman* (i.e. a corpus hyoidei with a foramen, a palatoquadrate lacking an ascending process and a simple articulation between the palatoquadrate, otic capsule and columella auris) as well as for Alligatoridae (i.e. the presence of an epiphanial foramen, an alar process and well-developed sphenethmoid commissures), useful for distinguishing clades at early phases of their ontogeny. In addition, the study highlights possible ecological and functional drivers of chondrocranial diversity. While the dominance of the nasal capsule in *Alligator mississippiensis* may represent an adaptation to densely vegetated habitats, enhancing olfaction and respiration, the prominence of the orbitotemporal region in *Crocodylus porosus* could support robust jaw adductor musculature for capturing large prey in open aquatic environments.

Although hatchlings remain anatomically close to embryos, post-hatching growth introduces species-specific modifications likely driven by shifts in diet, habitat or developmental rate (e.g. [77,78]), resulting in greater interspecific divergence than is apparent during embryogenesis. Thus, chondrocranial morphology offers valuable insights into early functional differentiation and ancestral morphology, but it must be interpreted with caution when extrapolating to adult forms. Our findings highlight the importance of integrating ecological, developmental and phylogenetic data to better understand cranial evolution in Crocodylia. Future studies should broaden taxonomic and ontogenetic sampling to enable more comprehensive and robust analyses.

## Data Availability

The three-dimensional reconstruction of the cartilaginous skull of *Caiman crocodilus*, along with chondrocranial measurements supporting this study, is available in the electronic supplementary material [79].
